# Optimizing fresh-frozen plasma transfusion in surgical neonates through thromboelastography: a quality improvement study

**DOI:** 10.1007/s00431-022-04427-6

**Published:** 2022-02-24

**Authors:** Genny Raffaeli, Nicola Pesenti, Giacomo Cavallaro, Valeria Cortesi, Francesca Manzoni, Giacomo Simeone Amelio, Silvia Gulden, Luisa Napolitano, Francesco Macchini, Fabio Mosca, Stefano Ghirardello

**Affiliations:** 1 Department of Clinical Sciences and Community Health, Università Degli Studi Di Milano, Milan, Italy; 2 Neonatal Intensive Care Unit, Fondazione IRCCS Ca’ Granda Ospedale Maggiore Policlinico, Via Francesco Sforza, 28 20122 Milan, Italy; 3 Department of Statistics and Quantitative Methods, Division of Biostatistics, Epidemiology and Public Health, University of Milano-Bicocca, Milan, Italy; 4 Pediatric Anesthesiology and Intensive Care Unit, Fondazione IRCCS Ca’ Granda Ospedale Maggiore Policlinico, Milan, Italy; 5 Department of Pediatric Surgery, Fondazione IRCCS Ca’ Granda Ospedale Maggiore Policlinico, Milan, Italy; 6 Neonatal Intensive Care Unit, Fondazione IRCCS Policlinico San Matteo, Pavia, Italy

**Keywords:** Blood products, Viscoelastic assay, Coagulation, Hemostasis, Bleeding, Clot

## Abstract

Fresh frozen plasma (FFP) is largely misused in the neonatal setting. The aim of the study is to evaluate the impact of a Thromboelastography (TEG)-based Quality Improvement (QI) project on perioperative FFP use and neonatal outcomes. Retrospective pre-post implementation study in a level-III NICU including all neonates undergoing major non-cardiac surgery before (01–12/2017) and after (01–12/2019) the intervention. In 2018, the intervention included the following: (1) Training on TEG, (2) Implementation of TEG, and (3) Algorithm for TEG-directed FFP administration in surgical neonates. We compared pre- vs post-intervention patient characteristics, hemostasis, and clinical management. Linear and logistic regression models were used to evaluate the impact of the project on main outcomes. We analyzed 139 neonates (pre-intervention: 72/post-intervention: 67) with a mean (± SD) gestational age (GA) 34.9 (± 5) weeks and birthweight 2265 (± 980) grams which were exposed to 184 surgical procedures (pre-intervention: 91/post-intervention: 93). Baseline characteristics were similar between periods. In 2019, prothrombin time (PT) was longer (14.3 vs 13.2 s; *p* < 0.05) and fibrinogen was lower (229 vs 265 mg/dl; *p* < 0.05), if compared to 2017. In 2019, the intraoperative exposure to FFP decreased (31% vs 60%, *p* < 0.001), while the pre-operative FFP use did not change. The reduction of intraoperative FFP did not impact on mortality and morbidity. Intraoperative FFP use was lower in the post-intervention even after controlling for GA, American Society of Anesthesiologists score, PT, and fibrinogen (Odds ratio: 0.167; 95% *CI*: 0.070, 0.371).

*   Conclusion*: The TEG-based QI project for the management of FFP during neonatal surgery reduced intraoperative FFP exposure.

**Table:** 

**What is Known:** *• PT and aPTT are poor predictors of bleeding risk in acquired neonatal coagulopathy, leading to likely unnecessary fresh frozen plasma (FFP) transfusion in the Neonatal Intensive Care Setting. * *• As neonatal hemostasis is a delicate balance between the concomitant reduction of pro- and anti-coagulants drivers, thromboelastography (TEG) is a promising alternative for coagulation monitoring.*	
**What is New:** *• The implementation of TEG, training, and shared protocols contributed to reduced intraoperative FFP use, which was not associated with increased mortality or bleeding events.* *• These findings inform future research showing that there is clinical equipoise to allow for larger studies to confirm the use of TEG in NICUs and to identify TEG cut-offs for transfusion practice.*

**Supplementary Information:**

The online version contains supplementary material available at 10.1007/s00431-022-04427-6.

## Introduction

Critically ill neonates are frequently exposed to blood products. After red blood cells, fresh frozen plasma (FFP) transfusion is the most frequent intervention, being around 10% of all admissions to the Neonatal Intensive Care Units (NICUs) [1, 2]. This rate increases among extremely premature infants at risk of hemorrhagic events [1].

Current neonatal transfusion guidelines consider the use of FFP for (1) active bleeding with associated coagulopathy defined by abnormal coagulation tests [Prothrombin Time (PT); Activated Partial Thromboplastin Time (aPPT) and fibrinogen] and (2) increased risk of bleeding associated with coagulopathy [2].

However, in clinical practice, it has been shown that about 60% of FFP transfusions in neonatology are not evidence-based. The main reasons for non-compliance to guidelines were represented by the use of FFP as volume expander, bleeding without coagulopathy, and septic newborns without bleeding or coagulopathy [3].

Furthermore, prolonged PT and aPTT are not predictors of increased bleeding risk in neonatal acquired coagulopathy, as these tests are mainly responsive to procoagulant factors. Indeed, even at the lowest gestational ages, the neonatal hemostatic balance is maintained by the concomitant reduction of both pro-and anti-coagulant drivers when clotting status is defined by global tests such as thromboelastography (TEG) and thrombin generation procedure [4–6]. TEG is a viscoelastic evaluation of the clot, from its formation to its lysis; it considers both cellular and plasmatic components of hemostasis, thus allowing a qualitative and dynamic analysis of the in vivo process [7, 8], with an acceptable clinical reproducibility in neonates [9]. We have defined TEG reference intervals for the very low birth weight (VLBW) and term infants at birth and over the first month of life [5]. TEG has been part of the hemostatic diagnostic workup in our NICU since 2018. Furthermore, we planned to implement TEG evaluation and multidisciplinary training. We shared transfusion algorithms to standardize the hemostatic management of surgical neonates exposed to increased bleeding risk and heterogeneous FFP transfusion practices.

Indeed, in pediatric and adult surgery, TEG has significantly reduced intra-operative FFP use while decreasing the risk of postoperative bleeding with a beneficial impact in terms of outcomes [10–13].

The purpose of this study is to evaluate the impact of a TEG-based Quality Improvement Project (here called intervention) on FFP transfusion of neonates undergoing surgery.

## Materials and methods

This single-center retrospective pre-post implementation study was conducted in a level III NICU based in an academic hospital. All procedures were performed in accordance with the Helsinki Declaration. The Institutional Review Board approved the study protocol (Comitato Etico Milano Area 2 — n° 36_2021). Due to the retrospective nature of the study, the need for informed consent from the parents has been waived by our Institutional Review Board.

### Quality improvement project

The project was based on a three-step intervention.

#### Step 1: training of the multidisciplinary team

The team included neonatologists and anesthesiologists, who routinely care for surgical neonates in the perioperative period. Education focused on neonatal hemostasis and diagnostic workup, including the basic principles of viscoelastic assays and evidence-based guidance for blood transfusion. Didactic sessions were accompanied by hands-on training on TEG 5000® (Hemoscope; Haemonetics, Niles, IL, USA) and Viscoelastic Coagulation Monitor (VCM®) System (Entegrion, Inc., Durham, NC), organized in small groups to acquire technical skills and traces’ interpretation clues, through the revision of real clinical cases.

#### Step 2: develop a multidisciplinary standard operating procedure (SOP) for blood products use in the surgical neonate

The team leader of this initiative (S. Gh.) developed an interdepartmental protocol based on current national and international guidelines [2, 14]. However, before this intervention, a specific procedure related to neonatal FFP transfusion management during the perioperative period was lacking. Therefore, decisions regarding the need and timing for FFP were at the discretion of the attending anesthesiologist, mainly based on the combined evaluation of (1) risk factors for bleeding, (2) active clinical bleeding, and (3) coagulopathy.

**Fig. 1:**
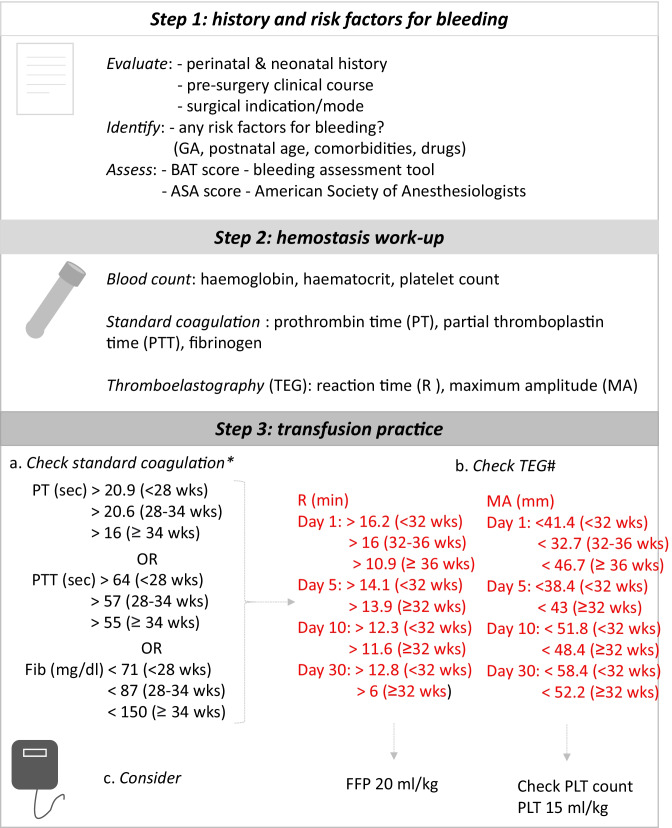
Diagnostic algorithm for the assessment of bleeding risk of the surgical neonate. *Reference ranges from Christensen et al. [15] Transfusion 2014; ^#^Reference ranges from Raffaeli et al. [5] Arch Dis Child Fetal & Neonat Ed 2020

During the post-intervention period, both neonatologists and anesthesiologists followed a structured approach for FFP administration (Fig. 1). Coagulopathy was defined as PT and PTT above the 95th percentile, and fibrinogen below the 5th percentile, based on gestational age-dependent reference ranges by Christensen et al. [15]. In case of abnormally prolonged PT and PTT, the decision to administer FFP was made according to TEG-Reaction Time (R) values above the 95th percentile, referring to our institutional TEG normative intervals [5]. The evaluation of TEG-Maximum Amplitude (MA) below the 5th percentile contributed to the definition of hypocoagulability (Fig. 1). VCM-Clotting Time (CT) and Maximum Clotting Firmness (MCF) express the same clinical significance of R and MA, respectively. Therefore, VCM data were referred to internal reference ranges for neonates.

The Hospital Transfusion Center supplied the FFP units. As for the internal procedure, based on SIMTI (Italian Society of Immunohematology and Transfusional Medicine) recommendations, we administered ABO/AB compatible FFP in aliquotes of 15–20 ml/kg [16].

#### Step 3: implementation of TEG in routine care in 2018

We have chosen 12 months of perioperative TEG use for three reasons: (1) to allow time to gain familiarity with the coagulation monitoring devices; (2) to avoid potential confounding factors, such as technical issues; (3) to rely on institution-based TEG references ranges for different gestational and postnatal age classes.

### Population

We retrospectively collected data from two cohorts of neonates exposed to major non-cardiac surgery born before (01/01/2017–31/12/2017) and after (01/01/2019 and 31/12/2019) the intervention.

We excluded patients with at least one of the following criteria: (1) congenital coagulopathy; (2) cardio-surgery, (3) minor surgery, (4) missing data related to perioperative transfusions.

We collected through the electronic patient charts Neocare® (GPI SpA), the following:

- Perinatal data: gender, gestational age (GA), birth weight, delivery mode, Apgar score, resuscitation;
- Pre- and intra-surgery data: American Society of Anesthesiologists score (ASA: grading of the anesthetic risk from 1-low to 5-high), hemostatic profile, blood products’ use, ventilatory and pharmacological management;
- Post-surgery outcomes: mortality, hemostatic, respiratory, and renal morbidity.

### Outcome measures

The main outcome measure was the number of neonates receiving FFP transfusion in the pre- and intra-operative period before and after the intervention.

As secondary outcomes, we evaluated across the two study periods: (1) the primary indications for FFP transfusion as reported in the patient charts; (2) the occurrence of abnormal coagulation defined as at least one prolonged PT or aPTT value exceeding 95°centile of the reference values [15]; (3) the number of thrombotic or major hemorrhagic events in the first 7 postoperative days; (4) the need for oxygen supplementation in the postoperative period compared to the pre-operative period; (5) onset of postoperative acute kidney injury (AKI) in the first 72 h after surgery; (6) need for intravenous diuretics in the 24 h following surgery; (7) postoperative weight gain; (8) mortality.

Bleeding (any site) was assessed through the NeoBAT score [17]. Cerebral hemorrhage was diagnosed by brain ultrasound and classified according to Papile et al. [18]. Major hemorrhage was defined as intraventricular hemorrhage ≥ grade 3, pulmonary hemorrhage defined as bleeding through an endotracheal tube with respiratory failure, and gastrointestinal hemorrhage defined as rectal bleeding.

AKI was defined as an increase in serum creatinine 24 h after surgery above 0.3 mg/dl with or without oliguria, diuresis rate below 1 ml/kg/h in the 24-h following surgery [19]. Diuresis was quantified as milliliters pro kilogram per hour; post-operative weight gain was estimated based on the increase in grams of post-operative daily weights (as a running total for postoperative days 0–5) referring to the pre-operative weight.

### Data analysis

Demographic and clinical parameters were presented through descriptive statistics. The comparison between the two periods was performed using *t*-test or Mann–Whitney U test for continuous variables and Fisher’s Exact test for categorical variables.

A logistic regression model was used to estimate the effect of the intervention on pre- and intra-operative FFP transfusions, mortality, the incidence of AKI, and hemorrhagic and thrombotic events. In addition, the impact on respiratory function was studied using a multiple linear regression model. All models were corrected for gestational age and clinical conditions. The results are presented in terms of odds ratio or regression coefficient, confidence interval, and *p*-value. All tests performed are two-sided, and a *p*-value < 0.05 is considered significant. Data analyses were performed using the R software, version 4.0.1 or higher (R Foundation for Statistical Computing, Vienna, Austria).

## Results

### Overall population

We analyzed 139 neonates (72 in pre-intervention; 67 in post-intervention) with a mean (± SD) GA of 34.9 (± 5) weeks and birthweight 2265 (± 980) grams, which were exposed to 184 surgical interventions (91 in pre-intervention; 93 in post-intervention). Urgent surgeries were 61% (56/91) in 2017 and 69% (64/93) in 2019. Baseline characteristics did not vary between periods (Table 1). In the post-intervention, PT was longer (14.3 vs 13.2 s; *p* < 0.05), and fibrinogen was lower (229 vs 265 mg/dl; *p* < 0.05) if compared to the pre-intervention. In 2019, intraoperative FFP transfusions decreased (31% vs 60%, *p* < 0.001), while the pre-operative FFP exposure did not change (Fig. 1, Table 2, Supplementary Tables S1–S2). Moreover, logistic regression showed a significant reduction in FFP intraoperative transfusions between 2017 and 2019 (*OR* = 0.275; 95% *CI*: [0.143; 0.517]; *p*-value < 0.001). This result is confirmed (Table 2) also adjusting for GA at birth and clinical conditions (*OR* = 0.167; 95% *CI*: [0.070; 0.372]; *p*-value < 0.001). The reduction of intraoperative FFP transfusion did not impact mortality and morbidity, except for a mild improvement of diuresis in the post-intervention (Table 2).

**Table 1:** Study population: demographic data and clinical outcomes (2017 vs 2019)

**Demographic data**	**2017 (*N*** **= 72)**	**2019 (*N*** **= 67)**	***p*-value**
Gestational age, weeks^a^	34.9 (4.6)	34.9 (5.2)	0.994^d^
Birthweight, grams^a^	2333.4 (950.6)	2402.8 (994.9)	0.675^d^
Apgar_5 minutes ^b^	9.0 (7.0; 10.0)	9.0 (8.0; 10.0)	0.672*
NICU lenght of stay^b^	27.5 (13.2; 55.5)	20.0 (10.5; 50.5)	0.444*
ASA_score^b^	4.0 (2.0; 4.2)	4.0 (3.0; 5.0)	0.724*
**Outcomes**			
Death^c^	8 (11.1)	7 (10.4)	> 0.999^e^
Bleeding post-surgery^c^	3 (4.2)	6 (9)	0.423^e^
Intraventricular hemorrhage^c^	2 (2.8)	4 (6)	0.612^e^
Thrombosis^c^	0	0	
Diuresis ml/kg/h ^a^	3.0 (1.6)	3.7 (2.1)	0.013^d^
Need for diuretics^c^	37 (40.7)	39 (42.4)	0.930^e^
Post-operative weight gain, grams^a^	99.8 (118.8)	94.7 (125.3)	0.794^d^
Acute kidney injury^c^	10 (11.0)	7 (7.6)	0.910^e^

Bleeding post-surgery was quantified based on NeoBAT score [17]

*ASA* American Society of Anesthesiologists’ score, *NICU* neonatal intensive care unit

*Mann–Whitney U test

^a^mean (SD)

^b^median (range)

^c^n (%)

^d^*t*-test

^e^Fisher’s exact test

**Table 2:** Linear and logistic regression models estimating the impact of the Quality Improvement Project, related to primary and secondary outcomes

		**Crude**			**Adjusted**^b^		
	Outcomes	Estimate	95% CI	*p*-value	Estimate	95% *CI*	*p*-value
Primary outcome	Preop FFP*	0.840	[0.364; 1.923]	0.678	0.653	[0.232; 1.756]	0.404
	Intraop FFP*	0.275	[0.143; 0.517]	** < 0.001**	0.167	[0.070; 0.372]	** < 0.001**
Secondary outcomes	Death*	1.017	[0.419; 2.489]	0.970	0.837	[0.306; 2.262]	0.725
	Death_7 days postop*	0.927	[0.212; 4.044]	0.917	0.718	[0.145; 3.471]	0.673
	Death_30 days postop*	0.923	[0.324; 2.633]	0.879	0.882	[0.283; 2.726]	0.825
	FiO2_preop^a^	0.027	[−0.031; 0.085]	0.363	0.021	[− 0.034; 0.076]	0.457
	FiO2_intraop^a^	1.673	[−1.526; 4.872]	0.302	2.585	[− 0.879; 6.049]	0.142
	FiO2_postop^a^	−0.042	[−0.097; 0.013]	0.130	−0.043	[− 0.097; 0.011]	0.115
	Acute kidney injury*	0.708	[0.242; 1.998]	0.515	0.604	[0.187; 1.862]	0.382
	Need for diuretics*	0.947	[0.509; 1.761]	0.863	1.010	[0.498; 2.052]	0.978
	Diuresis ml/kg/h^a^	0.505	[−0.003; 1.012]	0.051	0.641	[0.116; 1.165]	**0.017**

Estimate indicates OR or linear coefficient for logistic and linear regression respectively

*FFP*, fresh frozen plasma; *FiO2*, fraction inspired oxygen

*logistic regression

^a^linear regression

^b^Models have been adjusted based on gestational age, ASA score, PT, and fibrinogen values

### Intra-operative FFP use: comparison of transfused neonates 2017 vs 2019

**Fig. 2:**
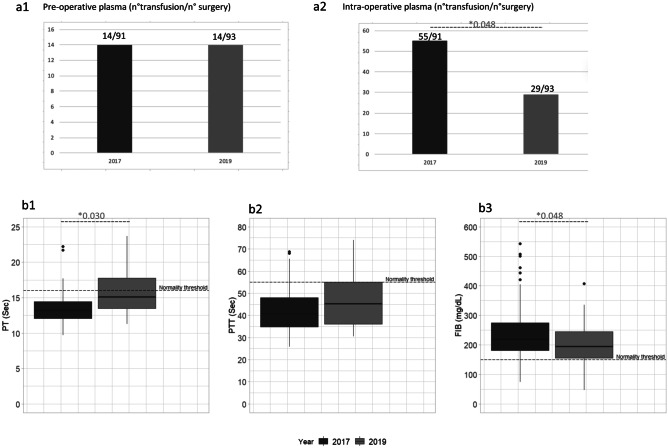
Fresh frozen plasma use (panel a): **a1**, pre-operative **a2**, intra-operative. Standard coagulation at the time of transfusion (panel b): **b1**, PT (seconds), **b2**, PTT (seconds) **b3** fibrinogen (mg/dl). *t-test

Drivers for intraoperative FFP prescription differed among study periods (Fig. 2, Table 3): in 2017, most transfusions (43.6%) were administered for volemic expansion, while coagulopathy was the main indication (58.6%) in 2019. Hypotension was reported as a determinant for FFP use only in the pre-intervention period. We could not retrieve the reason for FFP use in 27.3% and 10.3% of cases in the pre- and post-intervention period, respectively. If compared to the pre-intervention, PT was longer (15.8 vs 13.5 s; *p* < 0.003), postoperative platelet count (132 vs 205; *p* < 0.001) and fibrinogen were lower (203 vs 246 mg/dl; *p* = 0.048) in 2019. In the post-intervention, the neonates exposed to FFP transfusion had a mean TEG-R of 16 min and a mean TEG-MA of 51 mm. The clinical and hemostatic profile of neonates exposed to intraoperative FFP is shown in Table 3.

**Table 3:** Comparison of clinical determinants of intraoperative FFP administration

**Demographic data and outcomes**	**2017 (*N*** **= 45)**	**2019 (*N*** **= 25)**	***p*-value**
Gestational age, weeks^a^	34.7 (4.5)	32.9 (5.9)	0.155^d^
Birthweight, grams^a^	2250 (913)	2053 (1096)	0.424^d^
Apgar_5 minutes^b^	8 (7; 10)	8 (7; 9)	0.616*
NICU lenght of stay^b^	27 (14. 60)	33 (18. 92)	0.321*
Death^c^	8 (17.8)	5 (20.0)	> 0.999^e^
Post-operative bleeding^c^	3 (6.7)	5 (20.0)	0.198^e^
Intraventricular hemorrhage^c^	1 (2.2)	3 (12.0)	0.250^e^
Thrombosis^c^	0	0	
Acute kidney injury^c^	7 (15.6)	6 (24.0)	0.582^e^

**Perioperative hemostatic management**	**2017 (*N* = 55)**	**2019 (*N* = 29)**	***p*-value**
Pre-operative FFP^c^	9 (16.4)	10 (34.5)	0.098^e^
Red blood cells intra-op^c^	18 (32.7)	13 (44.8)	0.343^e^
Albumine intra-op^c^	1 (1.8)	0	> 0.999^e^
PT, sec^a^	**13.5 (2.5)**	**15.8 (3.2)**	**0.003**^d^
APTT, sec^a^	42.9 (10.6)	46.4 (12.4)	0.228^d^
Pre-op PLT, mmm3^a^	235.1 (151.4)	203.9 (95.3)	0.258^d^
Post-op PLT, mmm3^a^	**204.9 (119.0)**	**131.7 (73.0)**	**0.001**^d^
Fibrinogen, mg/dl^a^	**245.6 (102.8)**	**203.4 (80.3)**	**0.048**^d^
Reaction time R, min^a^	**-**	16.9 (4.6)	na
Maximum amplitude MA, mm^a^	**-**	51.0 (7.0)	na

**Reason for FFP use**	**2017 (*N* = 55)**	**2019 (*N* = 29)**	
Coagulopathy, *n* (%) (compliant to guidelines)	9 (16.4)	17 (58.6)	** < 0.001**
Volume expander, *n* (%) (not compliant to guidelines)	24 (43.6)	9 (31)	
Hypotension, *n* (%) (not compliant to guidelines)	7 (12.7)	0	
Missing data in the prescription record, *n* (%)	15 (27.3)	3 (10.3)	

Data related to the perioperative hemostatic management and reason for FFP use refer to the number of surgical interventions

*APTT* activated partial thromboplastin time, *na* not applicable, *NICU* neonatal intensive care unit, *PLT* platelet count, *PT* prothrombin time

*Mann–Whitney U test

^a^mean (SD)

^b^median (range)

^c^n (%)

^d^*t*-test

^e^Fisher’s exact test

### Intra-operative FFP use: comparison of non-transfused neonates 2017 vs 2019

In the pre-intervention, 36 out of 91 surgical interventions did not require FFP use. Of those, only one patient presented with a slightly prolonged aPTT, while the remaining neonates had a normal hemostatic profile. In the post-intervention, in 64 out of 93 surgical interventions, FFP was not necessary. The majority (55/64; 86%) had normal standard coagulation tests, while 9 out of 64 had prolonged PT or APTT. Among them, 8 out of 9 neonates had a normal TEG trace.

### Subgroup and multivariate analysis

The comparison of transfusion management between pre- and post-intervention by stratifying patients based on GA (< 34 vs ≥ 34 weeks) and ASA score (< 4 vs ≥ 4) led to the main results from the overall population (Supplementary Table S3).

## Discussion/conclusion

This study found that the Quality Improvement project based on TEG, staff training, and transfusion algorithm improved the hemostatic management of surgical neonates by reducing intraoperative FFP and supporting a more evidence-based FFP prescription. In addition, the reduced transfusion usage appeared to be safe as it did not impact the length of stay, mortality, bleeding events, and morbidity rates.

To our knowledge, this is the first attempt to improve FFP transfusion practice through a combined approach based on the viscoelastic assay, neonatal hemostatic training, and shared algorithms.

FFP is commonly administered in the NICU and, similarly to other blood products, it may be associated with (un)known risks, such as allergy, infections, transfusion-related acute lung injury (TRALI), and transfusion-associated cardiac overload (TACO) [20].

Indeed, under-estimation of transfusion-related adverse events is a major issue in neonatology, as transfused patients are usually sick. Data from UK hemovigilance showed that neonates are at higher risk of transfusion-related side effects than older children or adults [21]. Despite the availability of guidelines for neonatal blood products administration, adherence is still low for FFP, which is often administered prophylactically for prolonged PT and aPTT [3, 22].

The establishment of age-related reference ranges for standard coagulation tests and the shift from a routine admission coagulation screening to a clinically-oriented approach led to reduced FFP use [15, 23].

However, in selected neonatal categories, there is still a need for improvement in transfusion medicine. Surgical neonates illustrate this concept, as they usually undergo a coagulation screening before major procedures. The common multidisciplinary approach to these patients may include professionals that may be less familiar with neonatal hemostatic peculiarities.

In this context, the implementation of TEG, training, and shared transfusion algorithms increased compliance with FFP guidelines. Indeed, by comparing the reason for FFP transfusion across the two study periods, we may speculate a beneficial effect of training on a pre-existing knowledge gap in neonatal transfusion medicine. Furthermore, after the intervention, the number of transfusions with an off-guidelines indication declined, likely reflecting the increased awareness of healthcare providers of shared transfusion triggers. Nevertheless, our findings suggest that compliance with current guidelines can be further optimized.

In our opinion, the lack of effect of the intervention on the pre-surgery FFP use may rely on a “perceived” risk of bleeding in the most preterm, critical, and younger neonates. Indeed, Apgar scores, and gestational and postnatal age were lower and ASA scores higher in the pre-surgery transfused group, thus supporting this hypothesis.

The comparison of not-transfused neonates across the two study periods suggests a possible role for TEG. Exception made for those neonates presenting with normal standard coagulation tests, those with a prolonged PT or aPTT and a normal TEG trace did not receive FFP. We may speculate that in those cases, the attending physician was reassured by a normal viscoelastic test.

Moreover, the lack of effect of the reduced FFP use on mortality and primary morbidity outcomes may confirm the concept that FFP does not have a prophylactic effect in patients at higher risk of bleeding [15].

The improvement of diuresis observed in the post-intervention, although in the normal range in both study periods, further supports the concept that FFP transfusion may worsen cardiac and respiratory performances, namely TACO and TRALI [20, 24].

Indeed, FFP use has been identified as an independent risk factor for hemodynamically significant patent ductus arteriosus in preterm neonates due to a fluid management perturbation [25].

Interestingly, in this context, the reduction of FFP transfusion may partly reflect the increased awareness of neonatologists related to the emerging evidence that restrictive transfusion practices for red blood cells and platelets are beneficial, or at least non-inferior to liberal practices [26–28]. Nevertheless, the evidence for neonatal FFP use is still limited, and this study may provide a practical framework for resource optimization.

Potential limits require consideration. The study’s retrospective nature did not allow us to retrieve either the indication for intraoperative FFP transfusion in 18 out of 84 neonates or the eventual wastage of blood products. In addition, as this is a single-center project, the applicability of our findings to other NICUs may be somewhat limited. Furthermore, in our setting, the pre-intervention misuse of FFP was relevant, and this should be taken into account while evaluating the impact of the intervention itself. Lastly, although TEG and VCM are different devices, we deem our clinical experience adequate to evaluate the impact of this diagnostics on FFP use, further supported by institutional ranges.

In conclusion, our findings may inform future research showing that there is clinical equipoise to allow for larger studies to confirm the use of TEG in NICUs and to identify TEG cut-offs for transfusion practice. The implementation of viscoelastic assays combined to hemostatic training and shared algorithms should be encouraged to improve neonatal outcomes.

## Supplementary Information

Below is the link to the electronic supplementary material.

Supplementary file1 (DOCX 846 KB)

## Data Availability

All data generated or analyzed during this study are included in this article. Further inquiries can be directed to the corresponding author.

## Abbreviations

aPTT: Activated partial thromboplastin time
ASA: American Society of Anesthesiologists score
CT: Clotting time
CFT: Clotting formation time
FFP: Fresh frozen plasma
GA: Gestational age
MA: Maximum amplitude
NICU: Neonatal intensive care units
PT: Prothrombin time
QI: Quality improvement
R: Reaction time
TEG: Thrombo-elastography
TACO: Transfusion-associated cardiac overload
TRALI: Transfusion-related acute lung injury
VLBW: Very low birth weight
