# Supplementary material for: Optimizing fresh-frozen plasma transfusion in surgical neonates through thromboelastography: a quality improvement study
Source: Eur J Pediatr. 2022 Feb 24;181(5):2173–82. doi: 10.1007/s00431-022-04427-6 (PMC9056479; doi:10.1007/s00431-022-04427-6)

| **Supplementary Table 1. Perioperative hemostatic management** | | |  |
| --- | --- | --- | --- |
| **Perioperative hemostatic management** | **2017 (N=91)** | **2019 (N=93)** | **p.value** |
| Pre-operative FFP, ¶ | 14 (15.4) | 14 (15.1) | >0.999^ |
| Intra-operative FFP, ¶ | **55 (60.4)** | **29 (31.2)** | **<0.001^** |
| Intra-operative red blood cells, ¶ | 21 (23.1) | 24 (25.8) | 0.796^ |
| Albumine intra-op si, ¶ | 2 (2.2) | 7 (7.5) | 0.182^ |
| PT_sec, # | **13.2 (2.3)** | **14.3 (3.1)** | **0.011°** |
| APTT_sec, # | 41.9 (10.3) | 42.6 (11.4) | 0.671° |
| Pre-op PLT, mmm3 # | 282.1 (167.8) | 284.1 (149.8) | 0.933° |
| Post-op PLT, mmm3 # | 247.3 (146.4) | 212.9 (140.0) | 0.131° |
| Fibrinogen, mg/dl # | **264.9 (107.1)** | **228.8 (107.7)** | **0.029°** |
| Type of surgery_urgent, ¶ | 56 (61.5) | 64 (68.8) | 0.547^ |
| APTT: activated partial thromboplastin time; FFP: fresh frozen plasma; PLT: platelet count; PT: prothrombin time. # mean (SD), § median (range), ¶ n (%); °t-test, *Mann-Whitney U test, ^Fisher exact test. Data related to perioperative hemostatic management refer to the number of surgical interventions. | | | |
|  |  |  |  |
|  |  |  |  |
|  |  |  |  |

| **Supplementary Table 2. Clinical determinants of neonates exposed to pre-surgery FFP transfusion** | | | |
| --- | --- | --- | --- |
| **Variable** | **Yes (N=28)** | **No (N=156)** | **p.value** |
| Gestational age, mean (sd) | 32.3 (5.1) | 34.6 (5.0) | **0.032** |
| Birthweight, mean (sd) | 1943.6 (988.8) | 2291.9 (988.9) | 0.094 |
| Apgar 5 minutes, median (range) | 8.0 (4.0; 19.0) | 9.0 (4.0; 10.0) | **0.003** |
| Postnatal age, median (range) | 9.5 (1.0; 63.0) | 14.5 (0.0; 295.0) | 0.142 |
| Lenght of stay median (range) | 69.0 (3.0; 163.0) | 62.5 (2.0; 308.0) | 0.928 |
| NICU stay, median (range) | 35.0 (3.0; 124.0) | 32.5 (1.0; 236.0) | 0.539 |
| ASA_score, median (range) | 4.0 (2.0; 5.0) | 3.0 (2.0; 5.0) | **<0.001** |


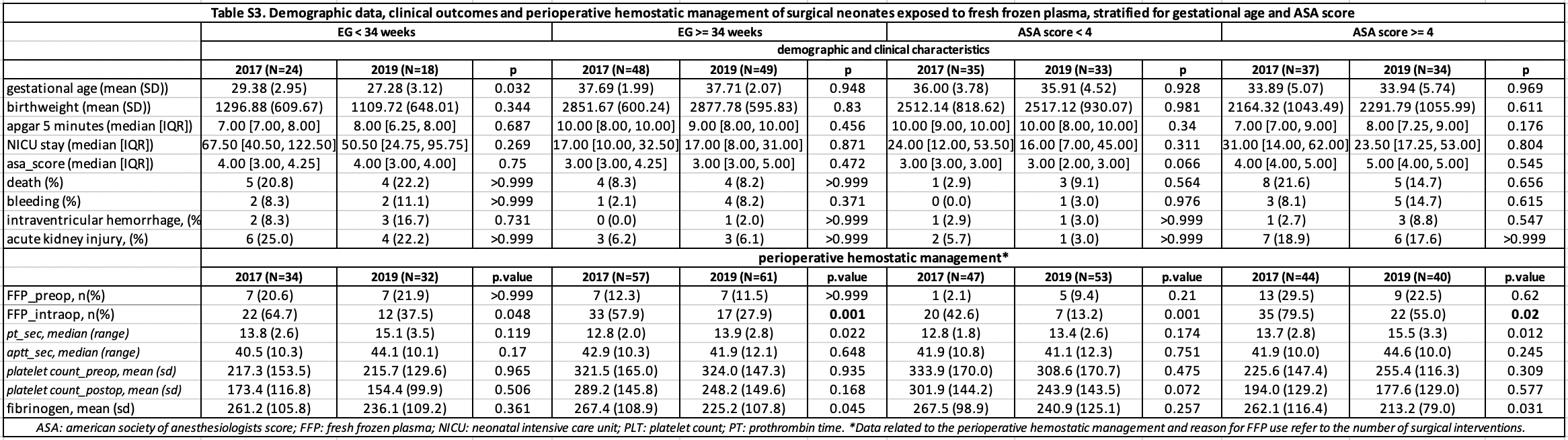

Supplement: Supplementary file 1 — Supplementary file1 (DOCX 846 KB) [file 431_2022_4427_MOESM1_ESM.docx]
